# Toll-like receptor 9 polymorphisms influence mother-to-child transmission of human immunodeficiency virus type 1

**DOI:** 10.1186/1479-5876-8-49

**Published:** 2010-05-25

**Authors:** Elisabetta Ricci, Sandro Malacrida, Marisa Zanchetta, Ilaria Mosconi, Marco Montagna, Carlo Giaquinto, Anita De Rossi

**Affiliations:** 1 Department of Oncology and Surgical Sciences, Oncology Section, AIDS Reference Center, University of Padova, Via Gattamelata 64, 35128 Padova, Italy; 2 Department of Neurosciences, University of Padova, Via Giustiniani 5, 35128 Padova, Italy; 3 Istituto Oncologico Veneto-IRCCS, Padova, Via Gattamelata 64, 35128 Padova, Italy; 4 Department of Pediatrics, University of Padova, Via Giustiniani 3, 35128 Padova, Italy

## Abstract

**Background:**

Toll-like receptors (TLRs) recognize pathogen-associated molecular patterns and play a crucial role in the host's innate immune response. Genetic variations in TLR genes may influence host-viral interactions and might impact upon the risk of mother-to-child transmission (MTCT) of Human Immunodeficiency Virus type 1 (HIV-1). The aim of this study was to investigate the influence of genetic variants of TLR 9 gene on MTCT.

**Methods:**

Three hundred children (118 HIV-1-infected and 182 HIV-1-uninfected) born to HIV-1-infected mothers were studied. Single nucleotide polymorphisms (SNPs) NM_017442.2: c.4-44G > A (rs352139) and c.1635A > G (rs352140) of the TLR9 gene were genotyped by TaqMan allelic discrimination assay. Statistical analyses were performed using SNPStats program.

**Results:**

When considered separately, neither of the two SNPs was significantly associated with risk of HIV-1 infection. However, the [A;A] and [G;G] haplotypes were associated with a higher risk of HIV-1 infection compared to the prevalent [G;A] haplotype [odds ratio (OR) = 3.16, 95% confidence interval (CI) 1.24-8.03, p = 0.016, and OR = 5.54, 95% CI 1.76-17.50, p = 0.004, respectively].

**Conclusions:**

Overall, results demonstrate a significant correlation between specific genetic variants of the TLR9 gene and risk of MTCT of HIV-1, thus confirming a critical role of innate immunity in perinatal HIV-1 infection. Strategies aimed at modulating innate immunity might be useful for future treatment of pediatric HIV-1 infection and AIDS.

## Background

Mother-to-child transmission (MTCT) is the main source of pediatric HIV-1 infection. MTCT of HIV-1 is multifactorial, with plasma viral load and mode of delivery being important maternal factors [1]. Innate immunity may contribute to host-viral interactions and impact the risk of MTCT of HIV-1 that occurs when the adaptive immune system is still under development.

Toll-like receptors (TLRs) are type 1 transmembrane proteins differentially expressed among immune cells. They recognize and bind to conserved pathogen-associated molecular patterns shared by large groups of microorganisms, and trigger the activation of signal transduction pathways, that in turn induce dendritic cell maturation and cytokine production [2]. These receptors play a central role in the activation of innate immunity [3]. A growing body of data supports a role of specific single nucleotide polymorphisms (SNPs) in several TLR genes in modulating the risk of bacterial and viral infections. Few studies have analyzed the role of TLR SNPs in clinical HIV-1 infection. An association between a TLR4 SNP and a higher susceptibility to tuberculosis in HIV-1-infected patients in Tanzania has been reported [4], and a functional TLR8 variant has been found to be associated with HIV-1 clinical outcome [5]. Recently, two SNPs in the TLR9 gene, the c.4-44G > A (rs352139) and the c.1635A > G (rs352140), were linked to progression of HIV-1 disease and viral load in adult patients [6-8]. TLR9 recognizes unmethylated cytidine-phosphate-guanosine (CpG) DNA motifs in bacteria and viruses [9], and is expressed in a wide variety of human cells, including plasmacytoid dendritic cells.

To date, the relationship between TLR gene polymorphisms and perinatal HIV-1 infection has not been investigated. The present study focuses on the role of c.4-44G > A and c.1635A > G SNPs in the TLR9 gene in MTCT of HIV-1 by analyzing a large cohort of HIV-1-infected and HIV-1-uninfected infants, all born to HIV-1-infected mothers.

## Methods

### Patients

The study population included 300 children born to HIV-1-seropositive mothers between 1984 and 1996, whose virological analyses for diagnosis of HIV-1 infection were conducted at the AIDS Reference Center of Padova University. Inclusion criteria were the known HIV-1-seropositive status of the mother at delivery, the absence of antiretroviral prophylaxis during gestation and/or at delivery, and ethnicity. All children were white Caucasian; 282 (94%) were born by vaginal delivery and 18 by caesarean section, and none was breastfeed. Diagnosis of HIV-1 infection was performed by virus isolation and polymerase chain reaction (PCR) [10]. For all children included in this study, 118 HIV-1-infected and 182 HIV-1-uninfected, the infection was confirmed by disease onset and/or persistence of HIV-1 antibody after 18 months of age, while lack of infection was confirmed by the loss of HIV-1 antibodies. The study was performed in accordance with the Helsinki Declaration and was approved by the Ethics Committee of the Azienda Ospedaliera di Padova. An informed consent regarding the use of biological specimens for research purposes was obtained from parents or legal guardians.

### SNP Analysis

Genomic DNA was extracted from peripheral blood mononuclear cells with the QIAamp DNA Blood mini kit (Qiagen, Hilden, Germany), according to the manufacturer's instructions. Polymorphic sites in genomic DNA were analyzed by TaqMan allelic discrimination assay. Primers and probes for NM_017442.2: c.4-44G > A (rs352139) and c.1635A > G (rs352140) genotyping were designed with Primer Express software (version 3.0, Applied Biosystems) (GeneBank reference sequence NM_017442.2). The primers were: 5'-CAGGTAGGGCTTGGAGAGAGG-3' and 5'-TGGGAGGGCTGTGTGAGTG-3' for c.4-44G > A and 5'-TGGACCTCTACCACGAGCACT-3' and 5'-AAAGGGCTGGCTGTTGTAGCT-3' for c.1635A > G. The TaqMan MGB allele-specific probes were: FAM-5' TGGAGGTGGAGCTG-3'-MGB and VIC-5'-TGGGTGGAGGTAGAG-3'-MGB for c.4-44G > A, FAM-5'-ACGGAGCTACCGCGA-3'-MGB and VIC-5'-CGGAGCTACCACGAC-3'-MGB for c.1635A > G. PCR was performed in the ABI PRISM 7700 thermal cycler (Applied Biosystems) according to standard procedures. Thermal cycling conditions were 2 minutes at 50°C, 10 minutes at 95°C, and 45 cycles each of 95°C for 15 seconds and 62°C for 1 minute. The genotypes were assigned using the Sequence Detection System software (version 1.9, Applied Biosystems), analyzing the threshold cycle of amplification curves. Accuracy of genotyping was confirmed by direct sequencing of randomly selected samples, as previously described [11], using the same primers used in Taqman assay.

### Quantification of HIV-1 RNA in plasma

Plasma HIV-1 RNA levels were determined by reverse transcriptase-PCR (Roche Amplicor Monitor System, New Jersey, USA) according to the manufacturer's instructions. The lower limit of detection of this assay was 50 HIV-1 RNA copies/ml of plasma using the ultrasensitive protocol.

### Statistical analysis

Estimation of the power of the study to detect an association between SNPs and HIV-1 infection was evaluated using the genetic power calculator available at http://pngu.mgh.harvard.edu/~purcell/gpc/. The study had 80% power to detect significant (p < 0.05) effects with odds ratio (OR) greater than 2 for the A allele of c.4-44G > A and G allele of c.1635A > G. Haplotype frequencies were estimated from genotype frequencies using the Expectation-Maximization algorithm coded into the SNPStats program [12]. All the statistical analyses, including the Hardy-Weinberg equilibrium test, linkage disequilibrium estimation, as well as the association of SNP genotypes and haplotypes with HIV-1 infection status, were performed using SNPStats software [12]. To increase the power of the study, four genetic models (co-dominant, dominant, recessive, and additive) were considered. Moreover, the effect of viral load on the association of SNPs and haplotypes with HIV-1 infection was tested with multivariate regression analysis, implemented in the SNPStats program. ORs and 95% confidence intervals (CI) were calculated for each group compared to the reference class (homozygous genotype for the prevalent allele); p values were derived from the Chi-square test. The Bonferroni correction of the significance level was applied to account for multiple testing.

## Results

To analyze the impact of c.4-44G > A and c.1635A > G SNPs on HIV-1 perinatal infection, a cohort of HIV-1-infected (n = 118) and exposed HIV-1-uninfected (n = 182) children was analyzed with different genetic models. Genotype and allele frequencies for the two SNPs were in Hardy-Weinberg equilibrium in both groups and were in agreement with frequencies reported in the NCBI database on the Caucasian population. c.4-44GA was the most frequent genotype in both HIV-1-uninfected and HIV-1-infected children, while c.1635AG was the most frequent genotype in HIV-1-uninfected children. c.4-44GG and c.1635AA genotypes had a higher prevalence in HIV-1-infected infants. However, the two SNPs showed no significant association with the risk of HIV-1 infection in the codominant model (Table 1), or in other genetic models considered (data not shown).

**Table 1:** Frequencies of TLR9 genotypes and haplotypes and risk of HIV-1 infection.

SNP	Genotypes	Uninfected children (n = 182)	Infected children (n = 118)	OR	95% CI		*p*
						
					LCL	UCL	
**c.4-44G > A(rs352139)**	GG	0.29	0.36	1			0.48
	GA	0.50	0.44	0.72	0.42	1.22	
	AA	0.31	0.20	0.80	0.42	1.53	
							
**c.1635A > G(rs352140)**	AA	0.31	0.39	1			0.20
	AG	0.48	0.38	0.62	0.37	1.06	
	GG	0.21	0.23	0.86	0.46	1.62	
							
**Haplotypes**	G;A	0.53	0.51	1			
	A;G	0.44	0.36	0.92	0.65	1.30	0.63*
	A;A	0.02	0.07	3.16	1.24	8.03	0.016*
	G;G	0.01	0.06	5.54	1.76	17.50	0.0038*

LCL, lower confidence limit; UCL, upper confidence limit.

* The significance level after Bonferroni correction for multiple testing is 0.017 instead of 0.05.

Linkage disequilibrium analysis confirmed a strong disequilibrium level between the two SNPs with D' = 0.87 and r^2 ^= 0.74. To investigate their combined effect, given their close proximity, haplotype frequencies were estimated in both groups of children. [G;A] was the most frequent haplotype in both HIV-1-uninfected and HIV-1-infected children, while the [G;G] and [A;A] haplotypes had a low frequency, but were more prevalent in HIV-1-infected infants, leading to a significantly increased risk of MTCT of HIV-1 compared to the most frequent [G;A] and [A;G] haplotypes (Table 1).

Values of maternal viral load at delivery were available in a subgroup of 109 infants (median 8790 HIV-1 RNA copies/ml of plasma, range 40-2106650 HIV-1 RNA copies/ml of plasma). Analyses of allele and genotype frequencies and the Hardy-Weinberg equilibrium in this subgroup were in agreement with those in the entire cohort (data not shown). For prevention of MTCT of HIV-1, highly active antiretroviral therapy was recently recommended for all women with HIV-1 RNA levels of ≥ 1000 copies/ml of plasma [13]. A further analysis was thus performed categorizing mothers according to low (< 1000 HIV-1 RNA copies/ml of plasma) and high (> 1000 HIV-1 RNA copies/ml of plasma) viral load. As for the larger cohort, while neither of the two SNPs was significantly associated with a risk of HIV-1 infection, the [G;G] haplotype remained associated with a higher risk of MTCT of HIV-1 after adjustment for maternal viral load (OR = 10.04, 95% CI 1.70-59.37, p = 0.012; p = 0.017 significance level after Bonferroni correction).

## Discussion

TLRs play a central role in innate immunity and a number of genetic association studies suggest that some TLR polymorphisms may be associated with susceptibility to different diseases. There is convincing evidence that common TLR SNPs modulate immune function by modifying the control of inflammatory cascades, elaboration of effector molecules, pathogen killing, and interactions with the adaptive immune response [14]. TLR9 plays a pivotal role in the induction of first-line defense mechanisms of the innate immune system and triggers effective adaptive immune responses to different bacterial and viral pathogens [15,16]. A few genetic polymorphisms within the TLR9 gene have been reported to be associated with a variety of inflammatory and infectious diseases [6-8,17,18].

Our study investigates for the first time the relationship between c.4-44G > A and c.1635A > G SNPs of TLR9 and MTCT of HIV-1, revealing that the TLR9 [A;A] and [G;G] haplotypes are associated with a significantly increased risk of MTCT of HIV-1. Genetic variations of TLR9, as a key gene of innate immunity, could impact on immunological downstream responses that are critically important for host defenses. The function of these SNPs and haplotypes is still largely unknown. Neither of the two SNPs induces amino acid change, but several findings suggest that they may affect TLR9 expression [7,8,17]. Functional studies demonstrated a critical role for the G allele of the c.4-44G > A SNP on TLR9 expression [17], and the c.4-44GG genotype was found to be associated with slow disease progression in HIV-1 infected adults [6]. The c.1635A > G SNP was also found to influence the progression of HIV-1 disease [6-8]. Furthermore, association of the c.1635A > G with CD4 cell count and viral load has suggested a role of this SNP in TLR9-mediated immune activation [7,8]. It has been demonstrated that sooty mangabeys have reduced levels of innate immune activation during apathogenic simian immunodeficiency virus infection and that plasmocytoid dendritic cells from these animals produce markedly low levels of interferon α in response to TLR9 ligands [19]. Specific haplotypes in the TLR9 gene might influence the functional ability of TLR9 to elicit a defense mechanism by affecting susceptibility or resistance to infections.

## Conclusions

In conclusion, our results demonstrate an important role of genetic variants of the TLR9 gene in modulating the risk of MTCT of HIV-1, thus confirming the relevance of innate immunity in perinatal HIV-1 infection. This knowledge may be valuable in the development of new therapeutic strategies including the use the specific adjuvants. More studies are needed to evaluate if strategies aimed at modulating innate immunity might be useful for future treatment of pediatric HIV-1 infection and AIDS.

## List of abbreviations

CI: confidence intervals; HIV-1: Human Immunodeficiency Virus type 1; MTCT: mother-to-child transmission; OR: odds ratio; SNP: single nucleotide polymorphism; TLR: Toll-like receptor.

## Competing interests

The authors declare that they have no competing interests.

## Authors' contributions

ER planned the study, performed genotyping and drafted the manuscript. SM performed statistical calculations and drafted the manuscript. IM contributed to genotyping and drafted the manuscript. MM contributed to statistical calculations and drafted the manuscript. MZ contributed to patient inclusion and handled samples collection and storage until nucleic acid extraction. CG recruited patients, collected clinical data, and contributed to study design. ADR planned and coordinated the study, supervised genotyping and statistical calculations, and drafted the manuscript. All authors read and approved the final manuscript.
